# Combating Antimicrobial Resistance Through a Data-Driven Approach to Optimize Antibiotic Use and Improve Patient Outcomes: Protocol for a Mixed Methods Study

**DOI:** 10.2196/58116

**Published:** 2024-11-08

**Authors:** Jonathan Mayito, Conrad Tumwine, Ronald Galiwango, Elly Nuwamanya, Suzan Nakasendwa, Mackline Hope, Reuben Kiggundu, Dathan M Byonanebye, Flavia Dhikusooka, Vivian Twemanye, Andrew Kambugu, Francis Kakooza

**Affiliations:** 1 Infectious Diseases Institute College of Health Sciences Makerere University Kampala Uganda; 2 African Center of Excellence in Bioinformatics and Data Intensive Sciences The Infectious Diseases Institute College of Health Sciences, Makerere University Kampala Uganda

**Keywords:** antimicrobial resistance, AMR database, AMR, machine learning, antimicrobial use, artificial intelligence, antimicrobial, data-driven, mixed-method, patient outcome, drug-resistant infections, drug resistant, surveillance data, economic, antibiotic

## Abstract

**Background:**

It is projected that drug-resistant infections will lead to 10 million deaths annually by 2050 if left unabated. Despite this threat, surveillance data from resource-limited settings are scarce and often lack antimicrobial resistance (AMR)–related clinical outcomes and economic burden. We aim to build an AMR and antimicrobial use (AMU) data warehouse, describe the trends of resistance and antibiotic use, determine the economic burden of AMR in Uganda, and develop a machine learning algorithm to predict AMR-related clinical outcomes.

**Objective:**

The overall objective of the study is to use data-driven approaches to optimize antibiotic use and combat antimicrobial-resistant infections in Uganda. We aim to (1) build a dynamic AMR and antimicrobial use and consumption (AMUC) data warehouse to support research in AMR and AMUC to inform AMR-related interventions and public health policy, (2) evaluate the trends in AMR and antibiotic use based on annual antibiotic and point prevalence survey data collected at 9 regional referral hospitals over a 5-year period, (3) develop a machine learning model to predict the clinical outcomes of patients with bacterial infectious syndromes due to drug-resistant pathogens, and (4) estimate the annual economic burden of AMR in Uganda using the cost-of-illness approach.

**Methods:**

We will conduct a study involving data curation, machine learning–based modeling, and cost-of-illness analysis using AMR and AMU data abstracted from procurement, human resources, and clinical records of patients with bacterial infectious syndromes at 9 regional referral hospitals in Uganda collected between 2018 and 2026. We will use data curation procedures, FLAIR (Findable, Linkable, Accessible, Interactable and Repeatable) principles, and role-based access control to build a robust and dynamic AMR and AMU data warehouse. We will also apply machine learning algorithms to model AMR-related clinical outcomes, advanced statistical analysis to study AMR and AMU trends, and cost-of-illness analysis to determine the AMR-related economic burden.

**Results:**

The study received funding from the Wellcome Trust through the Centers for Antimicrobial Optimisation Network (CAMO-Net) in April 2023. As of October 28, 2024, we completed data warehouse development, which is now under testing; completed data curation of the historical Fleming Fund surveillance data (2020-2023); and collected retrospective AMR records for 599 patients that contained clinical outcomes and cost-of-illness economic burden data across 9 surveillance sites for objectives 3 and 4, respectively.

**Conclusions:**

The data warehouse will promote access to rich and interlinked AMR and AMU data sets to answer AMR program and research questions using a wide evidence base. The AMR-related clinical outcomes model and cost data will facilitate improvement in the clinical management of AMR patients and guide resource allocation to support AMR surveillance and interventions.

**International Registered Report Identifier (IRRID):**

PRR1-10.2196/58116

## Introduction

Antimicrobial resistance (AMR) is among the greatest threats to global health and is predicted to become the leading cause of death by 2050 [1,2]. Mortality due to AMR will rise from 700,000 deaths to 10 million deaths annually if nothing is done to halt the current trends [3]. Indeed, in 2019, only 8 years from when these projections were made, 1.27 million deaths were attributed to AMR, higher than those due to HIV, malaria, and tuberculosis [2]. Moreover, 4.95 million deaths were associated with AMR [1]. If this trend continues unchecked, the world will confront a reality where many infectious diseases have “no cure and no vaccine” [4].

In addition, AMR has significant economic costs. The World Bank estimates that AMR could result in US $1 trillion in additional health care costs by 2050 and US $1 trillion to US $3.4 trillion gross domestic product losses per year by 2030 [5]. The impact of higher AMR is unlikely to be spread equally, with those more vulnerable likely to pay the highest price, as low-income countries suffer the biggest proportionate loss of population and economic output [4]. Low-income countries disproportionately bear the AMR burden [6]. For instance, the all-cause mortality in the review by the Antimicrobial Resistance Collaborators was highest in low-income countries: 27.3 per 100,000 in western sub-Saharan Africa (SSA) compared with 6.5 per 100,000 in Australasia [2].

Despite this worrying burden in sub-Saharan Africa, AMR data remain scant, and the systems to monitor and generate AMR data are underdeveloped. The region has a low Joint External Evaluation score of 53%, described as a voluntary, collaborative, multisectoral score to assess a country’s capacity to prevent, detect, and rapidly respond to public health risks whether occurring naturally or due to deliberate or accidental events [7]. Many of the countries in the region lack national action plans for AMR, alluding to AMR not being among their health priorities [8]. In contrast, some high-income economies of Europe and America had already instituted various regional and national AMR action plans as early as 2014 [9-12].

AMR is a natural phenomenon and tends to arise from enzymatic degradation, alterations in antimicrobial targets, or a change in membrane permeability to the antimicrobials, leading to a longer or no response to antimicrobial medicines [13,14].

Community-level drivers of AMR are diverse and enormous yet usually ignored in AMR control strategies. Among the antimicrobials, antibiotics are among the most abused due to their easier accessibility, lower cost, and relative safety compared with other antimicrobials [15]. Many people in the communities use nonprescription-based acquisition of antimicrobials from informal providers for both human and animal use [16]. Such providers are usually poorly trained, leading to wrong drug-pathogen matching, and are likely to be motivated by financial gains to offer suboptimal doses or stock substandard antimicrobials [17-19]. Drugs disposed of in this way find their way into the environment, interact with pathogens, and promote development of AMR in the environment [20]. There is also widespread use of antimicrobials in food-producing animals to treat and prevent disease or promote growth [21]. AMR drivers among European countries include human ambulatory consumption of antibiotics and per capita expenditure on health, accounting for 74% of AMR variation [19], accelerated by the misuse and overuse of antimicrobials; poor infection prevention and control; limited access to quality affordable medicines, vaccines, and diagnostics; lack of awareness; and poor enforcement of prescription regulations [1]. Overall, the drivers of AMR are similar across various World Health Organization (WHO) regions. Infections due to resistant microbes are more difficult to treat, spread more, and are of higher severity, resulting in increased morbidity, mortality, and cost of health care.

Antimicrobial misuse and overuse are the most critical drivers of AMR. For example, a review of Slovenia’s surveillance data showed that the prevalence of invasive *Streptococcus pneumoniae* resistant to penicillin decreased by 47.1% following a 32.8% decline in the area under the receiver operating curve (AUC) [22]. In the same study, high consumption of clarithromycin resulted in the selection and predominance of macrolide-resistant *Streptococcus pneumoniae*. Therefore, given the little global effort to develop new antimicrobials, the control of misuse and overuse presents the best chance to tackle AMR, more so in resource-limited settings.

AMR is not without consequences to the individual and health systems; for example, the impact of methicillin-resistant *Staphylococcus aureus* on the management of hospital-associated infections with severe complications, prolonged hospital stays, mortality, and morbidity reduces the clinical and economic outcomes for patients [23-26].

Treatment of AMR infections necessitates the use of more expensive and potentially toxic medications, results in longer hospital stays, and ultimately increases the cost of treatment [1]. In addition, resistant infections pose a risk to specialized medical procedures—such as organ transplants, surgical operations, and cancer chemotherapy—denying many of these critically needed services [1].

Grudlewska-Buda et al [27] described the rise of AMR in major foodborne pathogens (*Campylobacter* spp., *Salmonella* spp., *Escherichia coli*, and *Listeria* monocytogenes) associated with the declining effectiveness of β-lactams, sulfonamides, tetracyclines, and fluoroquinolones and highlighting the risk of resistant zoonotic infections.

Although AMR is often framed as a “silent pandemic,” recent figures demonstrate that this is not accurate, especially considering that its mortality and morbidity are gradually increasing with the drivers of high antibiotic use in animals and humans [28,29].

Furthermore, disability-adjusted life years (DALYs) are also increasing, indicating a reduction in life expectancy and quality of life [30]. Despite the importance of these parameters in characterizing the AMR burden, they are not routinely available, hindering comprehensive efforts to control the spread and effects of AMR.

The proposed project therefore aims to close 4 important gaps in AMR surveillance and interventions: (1) improve access to AMR and antimicrobial use and consumption (AMUC) data; (2) describe the AMR and antibiotic prescribing patterns, changes in the patterns over time, and associated factors; (3) determine the economic burden of excessive antibiotic use and the effect on hospital budgets; and (4) develop a machine learning model to predict the clinical outcomes of bacterial infectious syndromes caused by resistant pathogens. We hypothesize that this study will contribute to AMR and AMUC surveillance, identification of priority antibiotics to target for stewardship interventions, determining the cost implications, and identifying characteristics of individuals more likely to have poor clinical outcomes related to AMR so as to guide clinical decisions and health system planning in the allocation of scarce resources.

The overall objective of the study is to use data-driven approaches to optimize antibiotic use and combat antimicrobial-resistant infections in Uganda.

The specific study objectives are to (1) build a dynamic AMR and AMUC data warehouse to support research in AMR and AMUC to inform AMR-related interventions and public health policy, (2) evaluate the trends in AMR and antibiotic use based on annual antibiotic and point prevalence survey (PPS) data collected at 9 regional referral hospitals over a 5-year period, (3) develop a machine learning model to predict the clinical outcomes of patients with bacterial infectious syndromes due to drug-resistant pathogens, and (4) estimate the annual economic burden of AMR in Uganda using the cost-of-illness approach.

## Methods

### Study Design

The study will use different study designs for each of the 4 specific objectives.

For objective 1, we will conduct a data curation project aimed at building an AMR and AMUC data warehouse from prior and future AMUC surveys and routine AMR surveillance data from patients with bacterial infectious syndromes.

For objective 2, we will use a retrospective study using annual antibiotic surveys (AAS) and PPS data to determine the trends in antibiotic use and AMR using data accrued over a 5-year period.

For objective 3, we will conduct a modeling study in which a machine learning model will be developed to predict the clinical outcomes of patients with bacterial infectious syndromes due to resistant pathogens.

For objective 4, we will conduct a prevalence-based, cost-of-illness, descriptive study to assess the economic burden of AMR in Uganda by determining the average cost per AMR case.

### Study Settings

**Figure 1:**
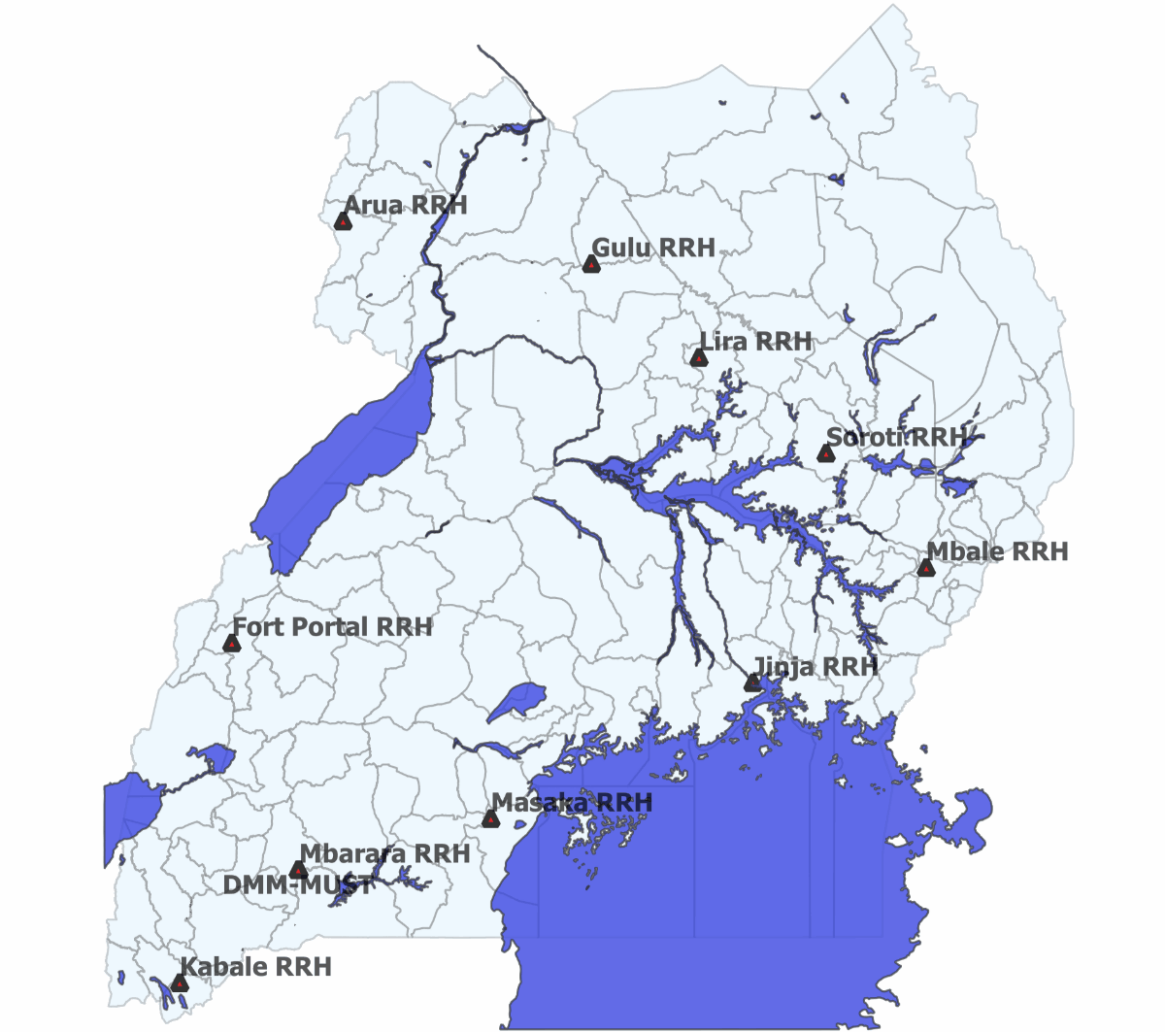
Geospatial representation of the human health antimicrobial resistance surveillance sites in Uganda. RRH: Regional Referral Hospital.

This study will use data already accrued under the Fleming Fund project or abstracted from clinical and other records at the Fleming Fund project–supported surveillance sites during the study period. The Fleming Fund Country Grant project (2018-2026) is a health system strengthening project to improve AMR surveillance at 9 regional referral hospitals (RRHs) in Uganda. The surveillance sites include Arua RRH, Gulu RRH, Lira RRH, Soroti RRH, Mbale RRH, Jinja RRH, Masaka RRH, Mbarara RRH, and Kabale RRH. The RRHs are spread across the different regions of the country (Figure 1) and are the first level of specialized health care in Uganda with specialist health workers and services.

### Study Population

The study will use records of patients with bacterial infectious syndromes attending the surveillance sites for health care.

#### Inclusion Criteria

All medical records of patients with bacterial infectious syndromes attending the major hospital wards including the medical, surgical, pediatric, gynecology, and maternity wards and the outpatient department are eligible for the study. Further, records related to medicines and supplies procurement as well as human resources and utility bills will be targeted by the study for the cost-of-illness evaluation.

#### Exclusion Criteria

For the AMR data, records without antimicrobial susceptibility test (AST) results will be excluded from the study.

### Study Procedures

#### Case Ascertainment

The patients with bacterial infectious syndromes will be identified from the diagnoses made by the attending clinicians and recorded in the patients’ clinical records. The resistant infections are determined from the AST results of the cultured samples showing resistance to at least one antibiotic.

#### Data Sources

Data will be abstracted from the procurement and human resources records and the clinical records of patients with bacterial infectious syndromes attending Arua RRH, Gulu RRH, Lira RRH, Soroti RRH, Mbale RRH, Jinja RRH, Masaka RRH, Mbarara RRH, and Kabale RRH.

#### Data Elements Collected

The treatment-related variables collected include referral status, age, gender, occupation, ward, residence, prior antibiotic treatment, date of admission, date of sample collection, sample type, diagnosis, and AST results. ASTs were performed using the Kirby-Bauer disc diffusion method according to the updated Clinical Laboratory Standards Institute M100, 30th, 31st, 32nd, and 33rd editions [31-34]. The AMUC variables collected include ward, number of prescribed antibiotics, number of prescriptions, number of injectable antibiotic prescriptions, prescriptions by generic name, prescriptions according to guidelines, prescriptions with appropriate diagnosis, cultures requested, prescriptions based on AST, missed doses, patients with missed doses, referrals, days spent on the ward, and number of hospitalizations in the last 90 days.

The clinical parameters to be collected will include temperature, blood pressure, level of antibiotic resistance, antibiotic type, diagnosis, prior antibiotic exposure, site of infection, duration of symptoms, timing of effective antimicrobial treatment, and medical comorbidities, while the clinical outcomes will include duration of hospital stay, time to clinical improvement, time to negative cultures, mortality, medical complications, disability, and the cost of treatment.

The cost-of-illness variables will include direct medical costs (personnel, medical supplies, drugs, laboratory tests, and patient out-of-pocket costs), direct nonmedical costs (recurrent expenditures such as utility bills), capital expenditures (such as expenditures on hospital or health facility infrastructure), patient transportation, and patient upkeep while seeking medical care. Indirect costs will include productivity losses due to illness-related absenteeism, reduced work hours, disability, or premature mortality, as well as informal care provided by family members or friends.

#### Data Abstraction Procedure

AMR data are collected during the routine care of patients with bacterial infectious syndromes at the surveillance sites. The process includes the identification of patients requiring sampling, drawing of the sample using appropriate techniques, transportation of the sample to the laboratory, sample integrity assessment, sample processing, recording of results on paper and in electronic data systems, and reporting of results for clinical management of the patients. On the other hand, AMUC data are collected through quarterly PPS and AAS. During PPS, data are abstracted from the records of all patients on the wards for at least 24 hours; AAS data are abstracted from 100 systematically selected records of patients seen at a particular ward in the last year. During PPS and AAS, data are collected from 6 hospital units including the outpatient department and medical, surgical, gynecology, pediatric, and maternity wards. The clinical outcome and cost-of-illness data will also be abstracted from the clinical, procurement, and human resource records alongside the PPS data over 3 quarters. The clinical outcome data will be collected from the 5 hospital units including the medical, surgical, gynecology, pediatric, and maternity wards.

The laboratory request form, laboratory results register, WHONET, and African Laboratory Information System are used to collect the AMR data, while AMUC data are collected using the WHO PPS tool and a standardized Microsoft Excel tool based on the WHO/ International Network for Rational Use of Drugs drug use indicator for the AAS data. The PPS tool is built in Open Data Kit (ODK), an Android app with offline capabilities installed on mobile smart devices including mobile phones and tablet computers and used for collecting, managing, and using data. The abstraction tool was pretested to evaluate the validation rules in ODK and to ensure the completeness, reliability, and validity of the tools. The AAS data are collected annually over a period of 2 weeks for each facility, while the PPS data are collected quarterly over a period of 2 days for each facility. The clinical outcome and cost-of-illness data will be collected quarterly using data abstraction forms built in REDCap [35].

Hospital pharmacists who are supervised by the senior pharmacists and doctors abstract the data after being trained on the data collection tools and the approach for collecting antimicrobial use (AMU) data. The supervisors review each completed form immediately after collection, and the finalized form is submitted to the server, after which it cannot be edited further. Appropriate use is assessed using the current Uganda Clinical Guidelines.

### Sample Size

#### Objective 1

All patients with bacterial infectious syndromes who access the surveillance sites for routine medical care are the primary source of the AMR data. The project has data on 12,366 patients over the last 4 years. We have also collected AMUC data on 13,500 patients over an 8-year period. We will also supplement these data with PPS data from 18,000 patients from AAS, giving a total of 31,500 patient records. As more data are gathered using the aforementioned data gathering techniques, the sample size will continue to increase for the periodic assessments.

#### Objective 2

The main outcome is the trends in the prevalence of antibiotic prescriptions. We used the following formula for sample size estimation for a proportion:

sample size = p(1-p)×(*z*/e)^2×n/[(1+(n-1))×r]

where *z* is the *z* score corresponding to the desired confidence level (in this case *z*=1.96 for a 95% CI), p is the estimated prevalence of antibiotic use (using 74% as the prevalence determined by Kiggundu et al [36] in 13 hospitals in Uganda), e is the margin of error expressed as a proportion (assumed to be 0.03), n is the number of clusters (in this case, the 9 surveillance sites), and r is the estimated intracluster correlation coefficient. The prevalence was similar across our surveillance sites; therefore, we used r=0.9 [36]. The sample size for each round of survey was determined as 0.74×(1-0.74)×(1.96/0.03)^2×9/((1+(9-1))×0.9) = 903.

Medical records of 100 patients from each of the major units including medical, surgical, pediatric, gynecology, and obstetrics/maternity wards and the outpatient department were selected for the AAS using systematic sampling. The nth patient was identified by dividing the total number of patients seen per year in each ward by 100 (the sample size). Therefore, 600 medical records were selected at each of the 9 surveillance sites during each survey, resulting in 27,000 (600×9×5) records in 5 years. On average, 20 to 30 records from each ward are included in the PPS, and 100 to 150 records from the 5 wards are considered for each of the 9 hospitals. Therefore 9000 (9×5×20×10) to 13,500 (9×5×30×10) records have been used for the last 10 PPS carried out to date. A total of at least 40,500 (27,000+13,500) records will provide the data for this evaluation. Therefore, the accrued data are sufficiently powered to estimate the prevalence and its trends over the evaluation period.

#### Objective 3

Generally, there is no fixed sample size for machine learning algorithms but the more data, the better the accuracy of the model at predicting the outcome. However, for prediction models, the rule of thumb for the sample size is to have at least 10 events for each predictor variable [37,38]. Therefore, the 18 variables that will be abstracted as outlined in the Data Elements Collected section would require at least 18×10=180 records. However, the study will utilize all available data from patients with bacterial infectious syndromes due to resistant and susceptible bacterial infections accrued through the quarterly data abstraction. We will use 70% of the data to train, while 30% of the data will be used to test the ability of the machine learning algorithm to predict the clinical outcomes of the patients with bacterial infectious syndromes.

#### Objective 4

Cost-of-illness or economic burden evaluations, unlike cost-effectiveness studies, are not typically based on a particular sample size [39]. The study will therefore utilize data from all patients with bacterial infectious syndromes due to resistance and those with susceptible bacterial infections and other related costs accrued through the quarterly data abstraction for this AMR economic burden evaluation.

### Statistical Analysis

#### Objective 1

##### Expected Outcome

The expected outcome is a refined and dynamic AMR and AMUC data warehouse that is linkable to other databases to enable collaborative research and programming for decision-making, policy formulation, and quality improvement projects.

##### Building the Data Warehouse

Building the data warehouse will comprise all data curation steps including cleaning, merging, cataloging, and integration. The data variables will be aligned to national and international AMR and AMUC indicators for easy alignment to contemporary literature. The curated data will be uploaded to a secure repository, which will be continuously updated as more data are accrued. The data repository will be housed in the African Centers of Excellence in Bioinformatics and Data Intensive Science (ACE) High-Performance Scientific Computing (HPC) IT infrastructure at the Infectious Diseases Institute (IDI). The HPC has 40 nodes, of which only 20 nodes are currently in use. The other nodes will be connected once demand increases. Each node has 32GB of RAM and 16 cores. It also has 120TB of Synology storage supported by a 30KVA inverter system that provides extra uptime in the event of a power outage and a monitoring system that monitors HPC systems for power surges and internet outages. It has a voltage stabilizer and batteries that last for up to 2 hours to 3 hours. Access to the HPC system is via a password-protected user account that is associated with an active email address, and all first-time users of the HPC must undergo a mandatory training session. Access to the servers will be via a Secure Shell (SSH) protocol to respective login nodes with access to compute nodes via the job scheduler (Slurm). User data directories and shared data directories will be backed up to enable recovery in the event of data loss.

The warehouse will adhere to the FAIR principles: (1) findable: where the data are easy to find by humans and computer and machine metadata standards and tags based on a defined criteria; (2) accessible: found data will be accessible through appropriate authentication and authorization; (3) interoperable: the accessed data would be interactable with other data sets through different applications and workflows for analysis, storage, and processing; and (4) reusable: the data will be well-indexed so that it can be replicated or combined with other data sets to interrogate wide research, surveillance, and stewardship questions.

Access to the data warehouse will be managed by role-based access control for the warehouse developers, where system administrators will assign user roles and manage access for each role. For the researchers and program investigators, the process to access the data in the warehouse will include submitting a data request form and the concept for intended use. The data request form will specify the type of data and the specific variables required. A scientific committee will review the concept and will recommend whether access should be granted. Before the data are accessed, the researcher or investigator will sign a data use agreement defining the terms under which the data will be used.

#### Objective 2

The objective 2 outcome variables will be derived as indicated in Table 1.

**Table 1:** Variables to assess objective 2 regarding antimicrobial use: measuring the impact on selected drug use indicators.

Indicator	Indicator definition		Disaggregation
	Numerator	Denominator	
Proportion of prescriptions with at least one antibiotic	Total number of antibiotic prescriptions	Total number of patients included in the sample	Gender of the patients Age of the patients
Proportion of prescriptions with an injectable antibiotic	Total number of antibiotic prescriptions with an injectable antibiotic	Total number of antibiotic prescriptions	Gender of the patients Age of the patients
Proportion of antibiotic prescriptions with a diagnosis that does not warrant an antibiotic	Number of antibiotic prescriptions with a diagnosis that does not warrant an antibiotic	Total number of antibiotic prescriptions	Gender of the patients Age of the patients
Proportion of antibiotic prescriptions for upper respiratory infections	Total number of antibiotic prescriptions for upper respiratory tract infections	Total number patients diagnosed with a URTI^a^	Gender of the patients Age of the patients
Proportion of antibiotic prescriptions^b^ in accordance with current treatment guidelines	Total number of antibiotic prescriptions in accordance with UCG^c^ 2016/2023 (antibiotic, dose, and frequency)	Total number of antibiotics prescriptions	Antibiotics prescribed by WHO^d^ AWaRe^e^
Proportion of antibiotic combinations with overlapping coverage	Total number of antibiotic combinations with overlapping coverage	Total number of antibiotic prescriptions	Antibiotics prescribed by WHO AWaRe

^a^URTI: upper respiratory tract infection.

^b^Prescription involving at least one antibiotic.

^c^UCG: Uganda Clinical Guidelines.

^d^WHO: World Health Organization.

^e^AWaRe: Access, Watch, Reserve.

Trend analysis will be used to derive the slope coefficient for the trends in the proportion of each antibiotic and level of AMR over a 5-year period for the 9 RRHs. The trends will be disaggregated by age, gender, bacterial infectious syndromes, wards, and bacteria types, among others.

#### Objective 3

##### Expected Outcomes

The expected outcome is a machine learning model to predict the clinical outcomes (length of stay, mortality, time to clinical improvement, mortality, and disability) of patients with bacterial infectious syndromes due to resistant pathogens using clinical parameters and demographics. The model will be deployed as a web or mobile device app-based physician assistant that can be used as a point-of-care aid in the management of patients with AMR.

##### Building the Machine Learning Algorithm

The abstracted data will routinely be curated and standardized; the diagnosis will be reported per the International Classification of Diseases 11 [40]. Several classification machine learning algorithms including logistic regression, artificial neural networks, support vector machines, random forest, and AdaBoost will be trained, and their performance will be evaluated by computing their accuracy, *F*_1_ score, precision, recall, and the AUC using a confusion matrix. The clinical outcomes of patients with bacterial-resistant infections will then be compared with those with susceptible infections using a chi-square test for categorical variables and nonparametric or parametric tests for the continuous data depending on the distribution of the data. The pipeline and derived algorithms will be stored on secure servers with access limited to only authorized personnel using the role-based access control model.

#### Objective 4

##### Expected Outcomes

The main outcome of this cost-of-illness analysis will be the cost per AMR case from societal and payer (government) perspectives.

##### Analysis of Outcome Variables

Data to parameterize the model will be obtained from the data warehouse and published gray literature. The activity-based (micro-costing) technique will be used to estimate the cost of diagnosis using the cost information in records related to the procurement of medicines, including antibiotics; AMR-related investigations; and clinical care of patients with AMR at the 9 surveillance sites.

The micro-costing technique decompounds each service (ie, diagnosis and treatment options) into the inputs and quantity required to provide it. The best price for each input will then be found and multiplied by the amount needed. The sum of all inputs provides a good estimate of the cost per intervention. Given that the analysis will be conducted from both the societal and payer (government) perspectives, direct medical costs, direct nonmedical costs, and indirect (productivity) costs will be included.

##### Estimating the Direct Medical Costs of AMR

To estimate direct medical costs, we will use the available databases of AMR data at IDI and the Ministry of Health (MOH). These data will aid in estimating the resource use and costs of identified AMR cases. Data on health resource use—drugs, laboratory tests, and other supplies—will be obtained from the literature to estimate the type and quantity of resources, which will then be multiplied by the unit costs obtained from the available price catalogs (from either Uganda’s Joint Medical Stores or Management Sciences for Health). Regarding personnel-related costs, we will use the opportunity cost of paid time for all health workers. Data on revised salaries will be obtained from the MOH or Ministry of Public Service to improve the precision of wage estimates.

All records related to the procurement of medicines including antibiotics, AMR-related investigations, and health care of patients with AMR at the 9 RRH surveillance sites over the 5-year period will be reviewed, and data will be abstracted. All the volumes and related costs of medicines procured, AMR-related investigations, and health care by the surveillance site over the evaluation period will be considered. All National Medical Stores invoices and delivery notes for each delivery cycle over the 5-year period will be reviewed, and data will be extracted for the volume of drugs delivered to the surveillance site, issued to the different units, and the unit cost of each medicine. In addition, information on clinical care including length of stay, investigations, and other medicines will be extracted. The parameters of interest will include volume and types of medicines and the cost of the medicines; types and cost of investigations; time of stay in the hospital; other costs of health care of patients with AMR, hospital medicines, investigations, and other sundries for clinical care; and total budget.

##### Estimating the Direct Nonmedical Costs of AMR

The overhead and recurrent costs related to outpatient and hospital treatment of AMR complications will be estimated from the available data in the MOH and IDI databases and the WHO-CHOICE (World Health Organization Choosing Interventions That are Cost-Effective) database for Uganda. All capital costs will be annualized using a discount rate of 3% with an assumed lifespan of 30 years for buildings, 5 years for computers, and 10 years for furniture to estimate the actual economic and opportunity costs. Other costs, such as transportation and upkeep for patients, will be obtained from the literature if unavailable at the MOH or IDI.

##### Estimating the Indirect Costs of AMR

Productivity losses for patients and their caregivers due to AMR-related morbidity and mortality will be estimated using the friction cost approach. The friction cost approach estimates the friction period—the time it takes an organization to replace an absent worker due to morbidity or mortality—and largely depends on the country’s unemployment rate [41,42]. We will sum up the lost time spent in transit to hospitals or health facilities (for patients and caregivers), seeking care, convalescing, and being admitted to a hospital (for patients and caregivers). The time lost will be valued at Uganda’s gross domestic product per capita as a proxy for wages, given the unavailability of these data in Uganda. The friction period accounts for absenteeism (actual absence from work) and presenteeism (available but not at the full productive level, thus affecting the workflow). Given Uganda’s unemployment rate of 12% [43], we will benchmark the published and gray literature to estimate the friction period for Uganda and indirect costs associated with AMR [42]. All future costs will be discounted at an annual rate of 3%, as recommended by the second panel on cost-effectiveness in health and medicine [44].

The societal cost will be the summation of direct medical costs, direct nonmedical costs, and indirect costs, while the payer cost will only include direct medical and direct nonmedical costs. The total cost for antibiotics, essential medicines, and other AMR-related health care costs will be derived from the product of the volume of the items consumed, duration of the provision of services, and the unit cost of each.

The main study outcome will be the cost per AMR case from societal and payer (government) perspectives. This will be the sum product of the quantity of resources needed to treat 1 AMR case and its unitary costs. All costs will be converted to US dollars using the bank of Uganda official exchange rates, while costs from the literature will be inflated to the reporting calendar year using the local consumer price indices. Given the ever-present uncertainty surrounding parameter estimates, we will assign different distributions to each model parameter using the 95% CIs and standard errors if available and, if unavailable, using a ±50% range. Monte Carlo simulation will be used to generate 1000 iterations of the model results; these new estimates will provide the cost of an average case of AMR with a 95% credibility range around the estimated cost. We will also perform a 1-way sensitivity analysis—presented as a tornado diagram—to determine which variables greatly influence costs. All analyses will be programmed in Microsoft Excel and supported by R software (version 4.3.0).

### Ethical Considerations

This study was approved by the IDI Research Ethic Committee (IDI-REC-2023-67:), which also granted a waiver of consent. The study was also approved by the Uganda National Council for Science and Technology (UNCST - HS3690ES). No participants’ identification information will be used in the dissemination or publication of the study results.

## Results

The study received funding from the Wellcome Trust through the Centers for Antimicrobial Optimisation Network (CAMO-Net) in April 2023. As of October 28, 2024, we completed data warehouse development, which is now under testing; completed data curation of the historical Fleming Fund surveillance data (2020-2023); and collected retrospective AMR records for 599 patients that contained clinical outcomes and cost-of-illness economic burden data across 9 surveillance sites for objectives 3 and 4, respectively.

## Discussion

This study will create a data warehouse and analyze AMR and AMUC rates and trends using data science and traditional statistical approaches. We will also determine the associated societal costs in Uganda and develop a machine learning model to predict AMR-related clinical outcomes. The dearth of data on AMU and AMR trends, clinical outcomes related to AMU and AMR, and the economic burden of AMR on the health care system is a major hinderance to policy formulation, stewardship interventions, and resource allocation.

The proposed data warehouse will help organize the AMR and AMUC data, making it accessible and linkable to other databases to allow deeper data mining to answer research and program questions through individual and collaborative research. This will contribute to one of WHO’s AMR surveillance plan pillars of generating knowledge through generation of quality data and evidence [45,46]. On the other hand, a concise description of AMR and AMU trends is critical to inform stewardship interventions to combat the escalating burden of AMR. Surveillance reports estimate that the AMR burden including mortality is disproportionately borne by SSA, but this has not been fully evaluated due to the scarcity of data in the region [47]. Further, optimization of AMU is important to address one of the biggest drivers of AMR in this setting—the overuse and misuse of antimicrobials [16,48,49]. By exploring AMU trends and the influencing factors, the study will inform strategies to address this problem.

Since the institution of the Global Antimicrobial Resistance and Use Surveillance System (GLASS) in 2015, Uganda, like many other countries in SSA, has developed its national action plan on AMR and started generating AMR and AMU surveillance data [50]. However, the AMR data generated lack information on certain aspects of the AMR burden including clinical outcomes (eg, disability and mortality) and the AMR economic burden [30]. Moreover, the few reports indicate that the DALYs for those who survive AMR are higher [51] and might equal to those of influenza, tuberculosis, and HIV combined [30]. This limits the optimization of clinical care for patients with AMR as well as national planning of AMR programs and guidance for resource allocation. This study will use machine learning algorithms and cost-of-illness estimation approaches to fill these critical gaps in the Uganda AMR surveillance data.

Our study’s strength lies in its focus on critical gaps currently limiting AMR surveillance in SSA and its multipronged approach to address the gaps including data science and economic evaluation approaches. This will provide information based on a wide and robust evidence base to inform AMR clinical management and control policies. The study is limited by the lack of a qualitative assessment of the gaps, which would have provided a comprehensive overview of the drivers of AMR and better informed the policy formulation process. Further, the use of retrospective data will pose challenges regarding missing variables and the inability to control for confounders for the observed outcomes, while the economic evaluation will impute some variables from the literature that are not routinely collected, which may bias some of the conclusions.

In conclusion, the data warehouse will promote access to AMR and AMU data to answer AMR program and research questions using a wide evidence base. The AMR-related clinical outcomes and AMR economic burden data will facilitate improvement in clinical management of patients with AMR and guide resource allocation to support AMR surveillance and interventions.

## Abbreviations

AAS: annual antibiotic surveys
ACE: African Centers of Excellence in Bioinformatics and Data Intensive Science
AMR: antimicrobial resistance
AMU: antimicrobial use
AMUC: antimicrobial use and consumption
AST: antimicrobial susceptibility test
AUC: area under the receiver operating curve
CAMO-NET: Centers for Antimicrobial Optimisation Network
DALY: disability-adjusted life year
FCA: friction cost approach
GLASS: Global Antimicrobial Resistance and Use Surveillance System
HPC: High-Performance Scientific Computing
IDI: Infectious Diseases Institute
MOH: Ministry of Health
ODK: Open Data Kit
PPS: point prevalence survey
RRH: regional referral hospital
SSA: sub-Saharan Africa
SSH: Secure Shell
WHO: World Health Organization
WHO-CHOICE: WHO Choosing Interventions That are Cost-Effective

## Data Availability

Data supporting this study are available from the IDI AMR Data Warehouse at [52]. Access to the data is subject to approval and a data sharing agreement due to legal or ethical reasons.
